# Magnetic resonance imaging findings in 11 cases of dedifferentiated endometrial carcinoma of the uterus

**DOI:** 10.1007/s11604-020-01084-3

**Published:** 2021-01-30

**Authors:** Nao Kikkawa, Kimiteru Ito, Hiroshi Yoshida, Mayumi Kobayashi Kato, Yuko Kubo, Yasuyuki Onishi, Haruto Sugawara, Tomoyasu Kato, Masahiko Kusumoto

**Affiliations:** 1 Department of Diagnostic Radiology, National Cancer Center Hospital, 5-1-1 Tsukiji, Chuo-ku, Tokyo, 1040045 Japan; 2 Department of Diagnostic Pathology, National Cancer Center Hospital, 5-1-1 Tsukiji, Chuo-ku, Tokyo, Japan; 3 Department of Gynecology, National Cancer Center Hospital, 5-1-1 Tsukiji, Chuo-ku, Tokyo, Japan

**Keywords:** Dedifferentiated endometrial carcinoma, MRI, Undifferentiated carcinoma, Endometrial carcinoma

## Abstract

**Purpose:**

We evaluated magnetic resonance imaging (MRI) findings of dedifferentiated endometrial carcinoma (DEC), comprising undifferentiated carcinoma and low-grade endometrioid carcinoma.

**Materials and methods:**

We recruited 11 patients with pathologically proven DEC treated at our institute. We evaluated primary lesion size, location and signal intensity on MRI, and prognosis. MRI and pathological findings were compared in eight resected patients.

**Results:**

Primary tumors ranged from 16 to 206 mm in diameter. DEC was located at the endometrium in 9 of the 11 patients; the remaining two patients showed diffuse involvement of the enlarged myometrium. These two patients with diffuse involvement type died within 4 months. Of the eight patients who underwent resection, seven had macroscopic intratumoral hemorrhage and six showed a high signal on T1-weighted images or low signal on T2-weighted images. Of the eight resected patients, four had tumor necrosis > 25% and tumor size > 5 cm. In these patients, necrosis appeared as nonenhanced areas on contrast-enhanced MRI.

**Conclusion:**

MRI findings of DEC showed two patterns: mass-forming type and diffuse myometrial type with poor prognosis. Most patients with DEC had intratumoral hemorrhage, and large tumors (> 5 cm) had gross necrosis, which appeared as nonenhanced areas on contrast-enhanced MRI.

## Introduction

Dedifferentiated endometrial carcinoma (DEC) is a rare and aggressive high-grade malignancy characterized by co-existence of undifferentiated carcinoma (UC) and low-grade endometrioid carcinoma (EmC) (grade 1 or 2 EmC). This carcinoma was reclassified by the World Health Organization 2014 Classification of Tumors of the Female Reproductive Organs [1]. In cases of DEC, the EmC and UC components coexist but are clearly separated from each other on histopathological examination. Therefore, DEC may be underestimated and diagnosed as EmC if needle biopsy alone is performed and the specimen is obtained from the low-grade EmC component. Silva et al. proposed that although DEC represents a gynecological tumor with different histological and clinical features from Grade 3 tumors classified according to the International Federation of Gynecology and Obstetrics (FIGO) [2], its clinical and pathological features may lead to misdiagnosis of FIGO Grade 3 EmC, lymphoma, or neuroendocrine tumor [3, 4]. Identification of the coexisting UC component and low-grade EmC and subsequent correct diagnosis is extremely important because this situation is associated with poor prognosis [4]. The gynecological symptoms of DEC are nonspecific (for example, postmenopausal bleeding) and can contribute to the difficulties in differentiating DEC from other endometrial carcinoma.

Most previous studies on DEC have described the clinical symptoms and pathological characteristics; Han et al. [5] reported pathological findings in four patients based on sagittal slice magnetic resonance imaging (MRI) or computed tomography (CT) images, although they did not focus on imaging findings.

However, so far, no case series have described the MRI findings and the correlation between radiological and pathological findings in DEC. This study aimed to correlate the MRI findings with the pathological findings in 11 patients with DEC.

## Materials and methods

The protocol for this retrospective study was approved by the institutional review boards of our institute (approval number: 2018-049); the study was conducted in accordance with the principles of the Declaration of Helsinki. The need for informed consent was waived due to the retrospective nature of the study.

### Participant recruitment

We recruited all consecutive patients with DEC of the uterus that was confirmed by pathology who were treated in our institution between May 1999 and May 2018. Cases of UC and Grade 3 EmC cases that were diagnosed between 1999 and 2016 underwent comprehensive pathological re-evaluation to identify missed cases of DEC. Specimens with inadequate volumes or those preserved under inadequate conditions were excluded. At least two board-certified pathologists reviewed hematoxylin–eosin-stained, formalin-fixed paraffin-embedded specimens to confirm the diagnosis and evaluate the histopathological findings of each case of DEC. Immunohistochemical staining of the following variant markers was performed where required for the diagnosis: epithelial differentiation makers; hormone receptors; DNA mismatch repair (MMR) proteins including MLH1, PMS2, MSH2, and MSH6 and core subunits of the switch/sucrose nonfermenting (SWI/SNF) complex, including ARID1A, ARID1B, SMARCA4, and SMARCB1 [6].

All participants underwent MRI with various 1.5 T or 3 T MRI scanners prior to treatment; we reviewed medical records and extracted data on clinical background including age, symptoms, clinical history, reproductive history, menopausal state, and histological procedures for diagnosis (needle biopsy or surgical resection). Additionally, FIGO staging and site of metastases were evaluated by reviewing pretreatment CT and/or positron emission tomography images. The primary outcome was defined as the date of death (whether disease-specific or not) or the date of the last documented visit.

### Magnetic resonance imaging protocol

The MR images were obtained at multiple referral hospitals using different scanners because of the rarity of DEC. Eight participants underwent MRI using a 1.5 T scanner; of them, five participants were scanned at external hospitals (using various scanners, namely Achieva [Philips Healthcare, Amsterdam, Netherlands], Syngo [Siemens medical solutions, Erlangen, Germany], Symphony [Siemens medical solutions], and Signa Excite [GE medical systems, Milwaukee, USA]) and three were scanned at our institute (using a VISART scanner [Canon Medical Systems, Tochigi, Japan], Syngo [Siemens medical solutions]). Three patients underwent MRI using a 3 T scanner (Achieva [Philips Healthcare] or Syngo [Siemens medical solutions]). All images were obtained using similar scan protocols with only minor variations between hospitals. Most MR sequences included the acquisition of T1- and T2-weighted images (WIs), diffusion-weighted images (DWIs) with b-values of 800–1000 s/mm^2^, and contrast-enhanced (CE) T1WIs. In general, gadolinium-based contrast agents were used for CE T1WI.

### Image interpretation

Each scan was reviewed by three board-certified radiologists (NK, YO, and HS), and findings agreed by consensus. The following imaging features were assessed: main occupied site (endometrium or myometrium), size, location, extent, and signal intensity of the tumor. Tumor size was measured as the maximum diameter of the tumor on sagittal T2WIs. For the mass-forming type, the main locations were divided into the upper, middle, and lower segments and the entire endometrial cavity. We classified the preoperative extent of the tumor in accordance with FIGO stages as superficial myometrial extension (with depth one-half less than that of the myometrium), deep myometrial extension (with depth one-half more than that of the myometrium), and parametrial extension based on MRI findings. In addition, the presence of cervical extension was assessed. The degree of signal intensity on T1WIs and T2WIs was classified visually as hypointense, isointense, or hyperintense by comparing it with that on the outer endometrium and myometrium. The homogeneity or heterogeneity of MR images of primary tumors was classified by the radiologists through visual assessment. The degree of tumor enhancement on CE-T1WIs was also classified visually as mild or strong by comparing with the normal myometrium.

### Pathological review

Each case was reviewed by one board-certified pathologist. Of the nine patients who underwent surgery, one was excluded because the tumor had resolved with preoperative chemotherapy; the remaining eight patients were evaluated macroscopically and microscopically. Intratumoral hemorrhage was classified as microscopically visible only (1 +) and macroscopically visible with area < 25% (2 +), 25–50% (3 +), and > 50% (4 +). Furthermore, necrotic areas inside the tumor were classified as microscopically visible only (1 +) and macroscopically visible with area < 25% (2 +), 25–50% (3 +), and > 50% (4 +). In addition, the approximate proportions of undifferentiated components within the tumors were visually assessed.

## Results

### Study population and clinical characteristics

In total, we recruited 11 participants for this retrospective study, which included eight (0.58%) of the 1386 resected cases of uterine tumors and three cases diagnosed by needle biopsy. Among the 11 cases, we re-evaluated eight surgical specimens and three needle biopsies for pathological diagnosis. The presence of both EmC and UC was confirmed from all surgical specimens; however, only UC was confirmed in needle biopsy specimens through morphological and immunohistochemical features. Because of the extensive sampling of surgically resected tumors at our institution, even in cases where the biopsy specimens contained only a UC component, all contained a small amount of low-grade EmC components in the hysterectomy specimens. Based on these experience, we took the position that the biopsy specimen alone could not determine DEC or UC. Therefore, we deemed DEC/UC to be inferred in biopsy specimens containing only UC components. No case had been diagnosed as pure UC in the resected cases of the 20 years we reviewed. In one case diagnosed by needle biopsy (no. 1), the surgical specimen after neoadjuvant chemotherapy (taxane and platinum-based chemotherapy) revealed no evidence of a residual tumor. Four patients received adjuvant chemotherapy (anthracycline and platinum-based chemotherapy) after radical surgery. The clinical features of the study population are summarized in Table 1. The median age was 54 years (range: 46–61). The most common symptoms were abnormal vaginal bleeding and abdominal pain. Six patients survived for a median of 80 months (range: 10–223 months) without recurrence, whereas five died at a median of 3 months (range: 3–18 months) from appearance of initial symptoms.

**Table 1:** Patient characteristics

No	Age (years)	Symptoms	Reproductive history	Menopausal status	FIGO staging	Site of metastases	Treatment	Procedures for diagnosis	Outcome	Observation period (month)
1	58	VB	0G0P	Postmenopausal	IVB	LN, peritoneum	NAC, Surgery	Needle biopsy*	Survive	29
2	54	Unknown	Unknown	Unknown	IB		Surgery	Surgical resection	Survive	223
3	50	VB	2G2P	Premenopausal	IIIC1	LN	Surgery, AC	Surgical resection	Survive	88
4	46	VB	0G0P	Premenopausal	IIIC1	LN	Surgery, AC	Surgical resection	Survive	84
5	59	VB	Unknown	Unknown	IB		Surgery	Surgical resection	Survive	76
6	48	VB, AP	1G1P	Premenopausal	IVB	LN, peritoneum, ovary	Surgery	Surgical resection	Death	3
7	52	VB	5G3P	Premenopausal	IIIA	ovary	Surgery, AC	Surgical resection	Survive	10
8	57	VB, AP	3G3P	Postmenopausal	IVB	LN, lung, adrenal gland	Palliative surgery	Surgical resection	Death	3
9	54	VB, AP	0G0P	Postmenopausal	IIIC2	LN	Surgery, AC, RT	Surgical resection	Death	18
10	61	VB, AP	2G2P	Postmenopausal	IVB	LN, peritoneum, pleura, bone	Palliative RT	Needle biopsy	Death	3
11	54	VB, AP	2G2P	Postmenopausal	IVB	LN, lung	Palliative RT	Needle biopsy	Death	4

*VB* vaginal bleeding, *AP* abdominal pain, *NAC* neoadjuvant chemotherapy, *AC* adjuvant chemotherapy, *LN* lymph node, *RT* Radiation therapy

*Surgical specimen of this case had no evidence of residual carcinoma as a result of NAC

### Magnetic resonance findings

The MRI findings of the study cohort are summarized in Table 2. Primary lesions of DEC showed two different patterns according to the shape of their development: mass-forming lesions arising from the endometrium with or without myometrial extension and diffuse myometrial involvement of the uterus without endometrial thickening.

**Table 2:** Correlation between MRI and pathological findings

No	Type of tumor growth	Size (mm)	Location	Myometrial extension	Cervical stromal extension	T1WI		T2WI		CE T1WI			DWI	Pathology		
						Intensity*	pHSI	Homogeneity	pLSI	Homogeneity	NEA	SEA		Hemorrhage	Necrosis	UC (%)
1	Mass forming	16	Upper	Absent	Absent	Iso	–	Homo	–				High			
2		29	Entire	Deep	Absent			Homo	+	Homo	–	–		2 +	2 +	20
3		36	Lower	Deep	Present	High	+	Homo	–	Hetero	–	+		2 +	1 +	70
4		40	Entire	Deep	Absent	Iso	–	Hetero	–	Hetero	–	+	High	2 +	2 +	90
5		47	Upper	Deep	Absent			Hetero	–	Homo	–	+		1 +	2 +	80
6		53	Middle	Deep	Absent	Iso	+	Hetero	–				High	3 +	4 +	95
7		67	Upper	Superficial	Absent	Iso	+	Hetero	+	Hetero	+	–	High	4 +	3 +	95
8		134	Entire	Deep	Present	Iso	+	Hetero	+	Hetero	+	–	High	2 +	4 +	99
9		151	Entire	Parametrium	Absent	Iso	+	Hetero	+	Hetero	+	–	High	2 +	4 +	90
10	Diffuse involvement	171	–	Parametrium	Present	Iso	+	Hetero	+	Hetero	+	–	High			
11		206	–	Parametrium	Present	Iso	+	Hetero	+				High			

*Deep* extension depth with more than one half of the myometrium, *Superficial* extension depth with less than one half of the myometrium

*Compared with the myometrium signal

*pHSI* Partially high signal intensity, *pLSI* Partially low signal intensity, *NEA* Nonenhanced area, *SEA* Strong enhanced area

1 + , Microscopic only; 2 + , Macroscopically < 25%; 3 + , 25–50%; 4 + , > 50%

UC (%), percentage of undifferentiated carcinoma compartment

**Fig. 1:**
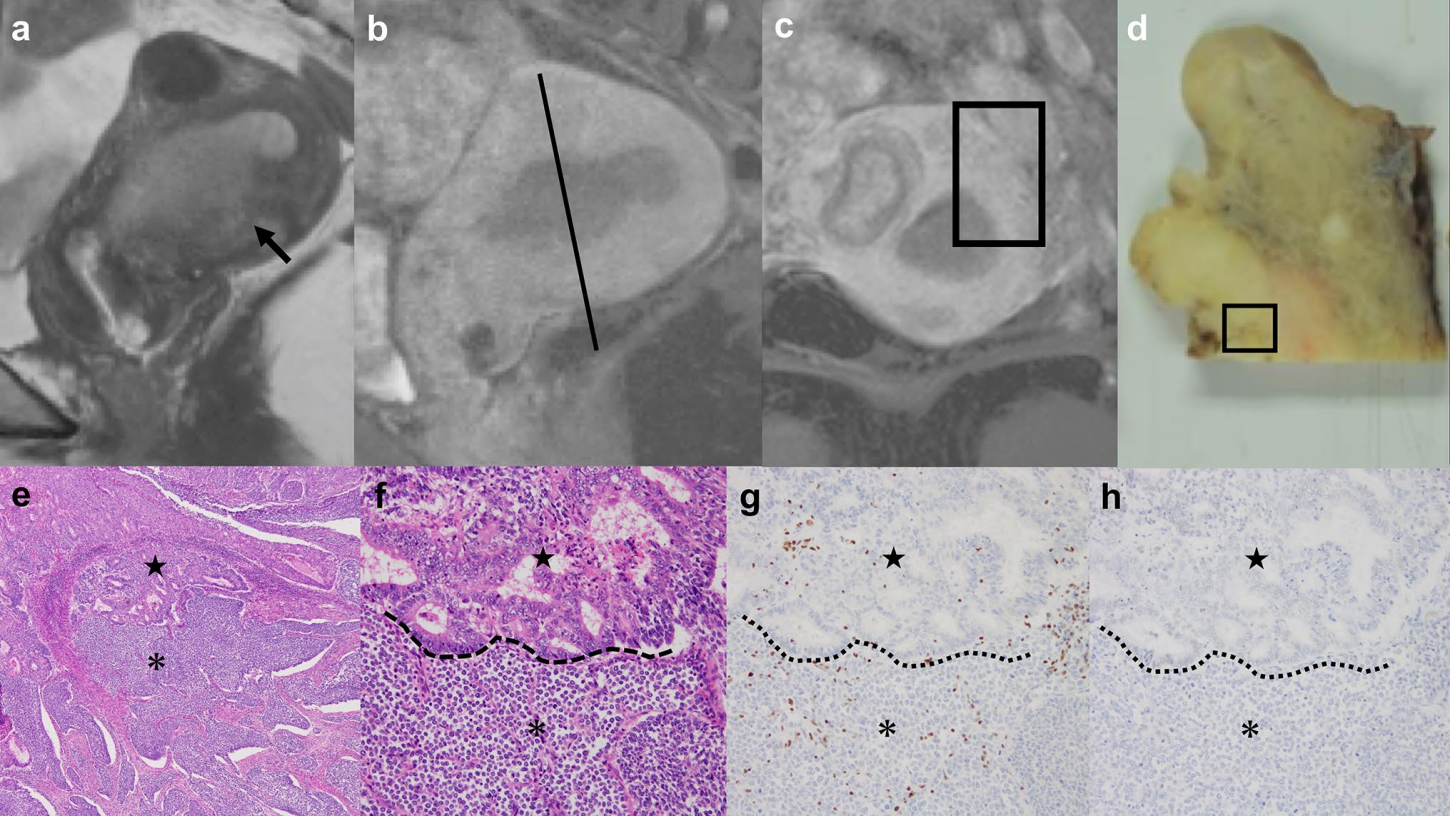
A case of a 46-year-old woman with dedifferentiated endometrial carcinoma (no. 4). **a** Sagittal T2-weighted image showing a mass-forming tumor located at the uterine with disruption of the junctional zone (arrow). **b** Sagittal contrast-enhanced T1-weighted image showing relatively homogeneous contrast enhancement with deep extension into the myometrium. **c** Coronal oblique contrast-enhanced T1-weighted image on black line in ‘**b**’. **d** Coronal oblique cut surface of the uterus enclosed by the square at ‘c’ showing a tumor in the endometrial cavity with extension into the myometrium. **e** Low-magnification image of dedifferentiated endometrial carcinoma in the black square in ‘d’ showing the borderline of undifferentiated carcinoma and low-grade endometrioid carcinoma (hematoxylin and eosin staining, 40 × magnification). Irregularly branched or fused tumor glands (indicated with ★) transition to solid tumor nests (indicated with *) and diffuse extension into the myometrium are observed. This distinction between undifferentiated and low-grade endometrioid carcinoma components was unclear on contrast-enhanced T1-weighted imaging. **f** Higher magnification images reveal the undifferentiated component (indicated with *) to be adjacent to the low-grade endometrioid carcinoma component (indicated with ★) (hematoxylin and eosin staining, 200 × magnification). The borderline between the components is clear (dotted line). Immunohistochemically, undifferentiated carcinoma cells show concurrent loss of **g **ARID1A (200 ×) and **h** ARID1B (200 ×) protein

**Fig. 2:**
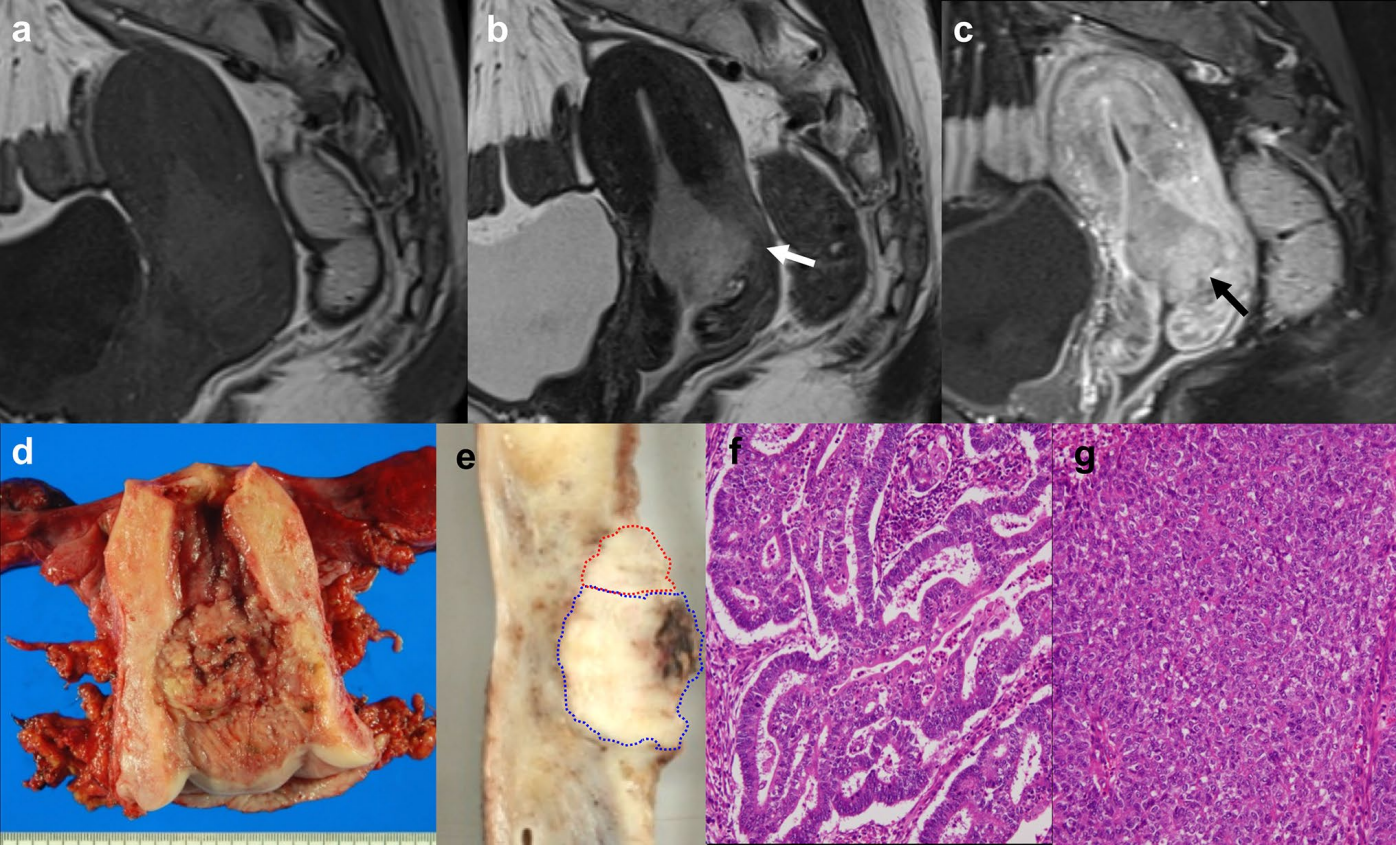
A case of a 50-year-old woman with dedifferentiated endometrial carcinoma (no. 3). **a** Sagittal T1-weighted image showing a mass-forming tumor located at the lower portion of the uterus. **b** T2-weighted image showing disruption of the junctional zone (white arrow). **c** Sagittal contrast-enhanced T1-weighted image showing heterogeneous enhancement with deep extension into the myometrium. Parts of the tumor show strong contrast enhancement after injection of the contrast agent (black arrow). **d** Gross pathology of the resected specimen showing exophytic tumor at the isthmus. **e** A sagittal cut surface of the uterus. The upper part is in the direction of the uterine fundus. Red and blue broken lines denote tumor areas containing low-grade endometrioid carcinoma and undifferentiated carcinoma, respectively. The strongly enhanced regions on contrast-enhanced T1-weighted image (‘**c**’) were approximately consistent with undifferentiated components. **f** Low-grade endometrioid carcinoma component. The tumor composed of columnar cells shows irregularly branched and fused glands (H&E, × 200). **g** Undifferentiated carcinoma component. The monotonous tumor cells glow in the sheet (H&E, × 200)

**Fig. 3:**
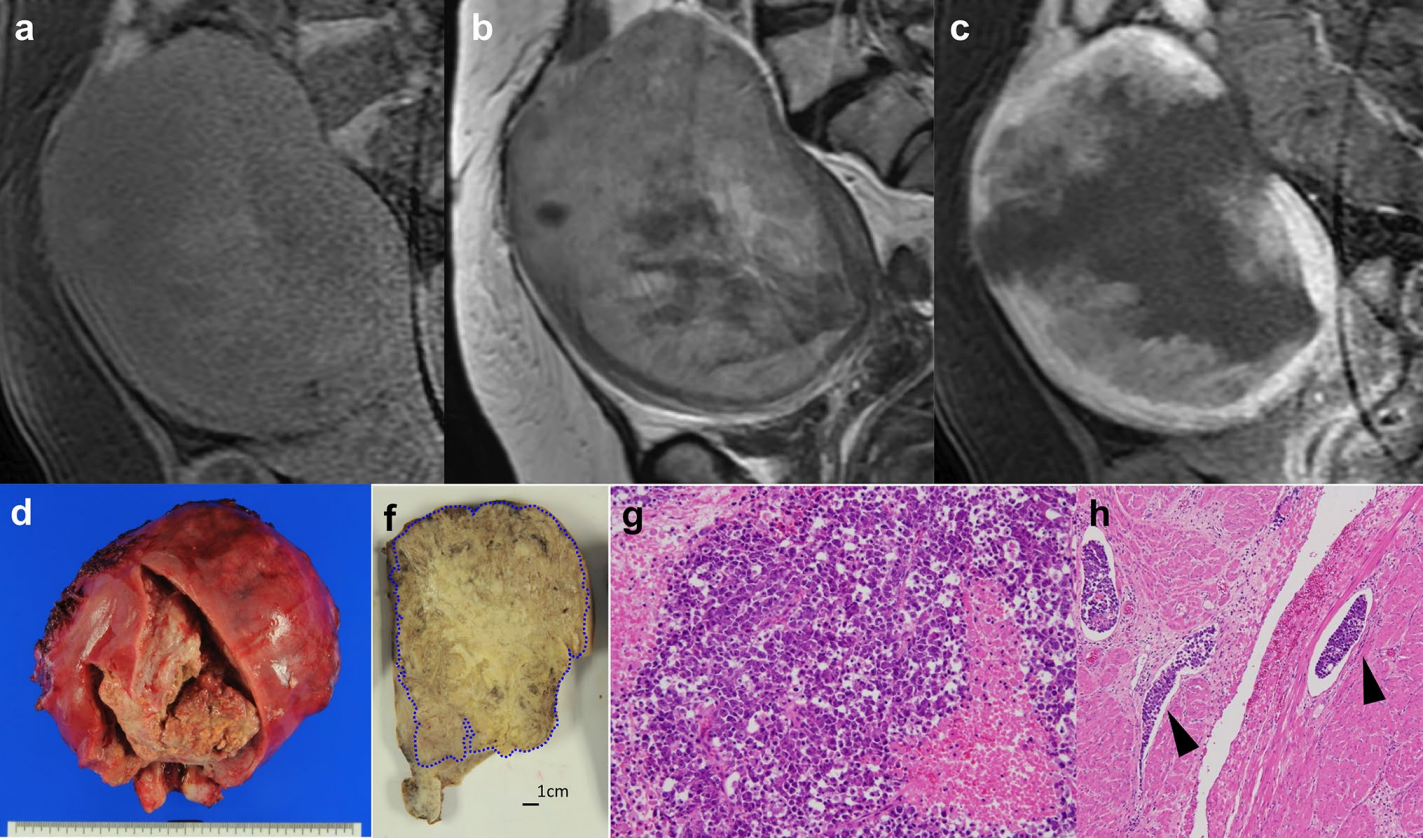
A case of a 57-year-old woman with dedifferentiated endometrial carcinoma (no. 8). **a** Sagittal T1-weighted image showing an enlarged uterus with an internal high signal intensity. **b** T2-weighted image showing a mass-forming tumor with deep infiltration of the myometrium. **c** Sagittal contrast-enhanced T1-weighted image showing extensive internal areas without tumor enhancement. **d** The uterus is enlarged, and a large necrotic tumor occupies the entire endometrial cavity. **e** A sagittal cut surface of the uterus. A large tumor has replaced the entire uterus and shows extensive areas of necrosis. Blue broken line denotes tumor area containing undifferentiated carcinoma. **f **Undifferentiated carcinoma. Tumor cells are monotonous and dyshesive without nest or gland formation. Prominent tumor cell necrosis (*) is observed (H&E, × 200). **g** There are numerous lymphatic involvements of the tumor (black arrow heads) at the peripheral myometrium (H&E, × 100)

**Fig. 4:**
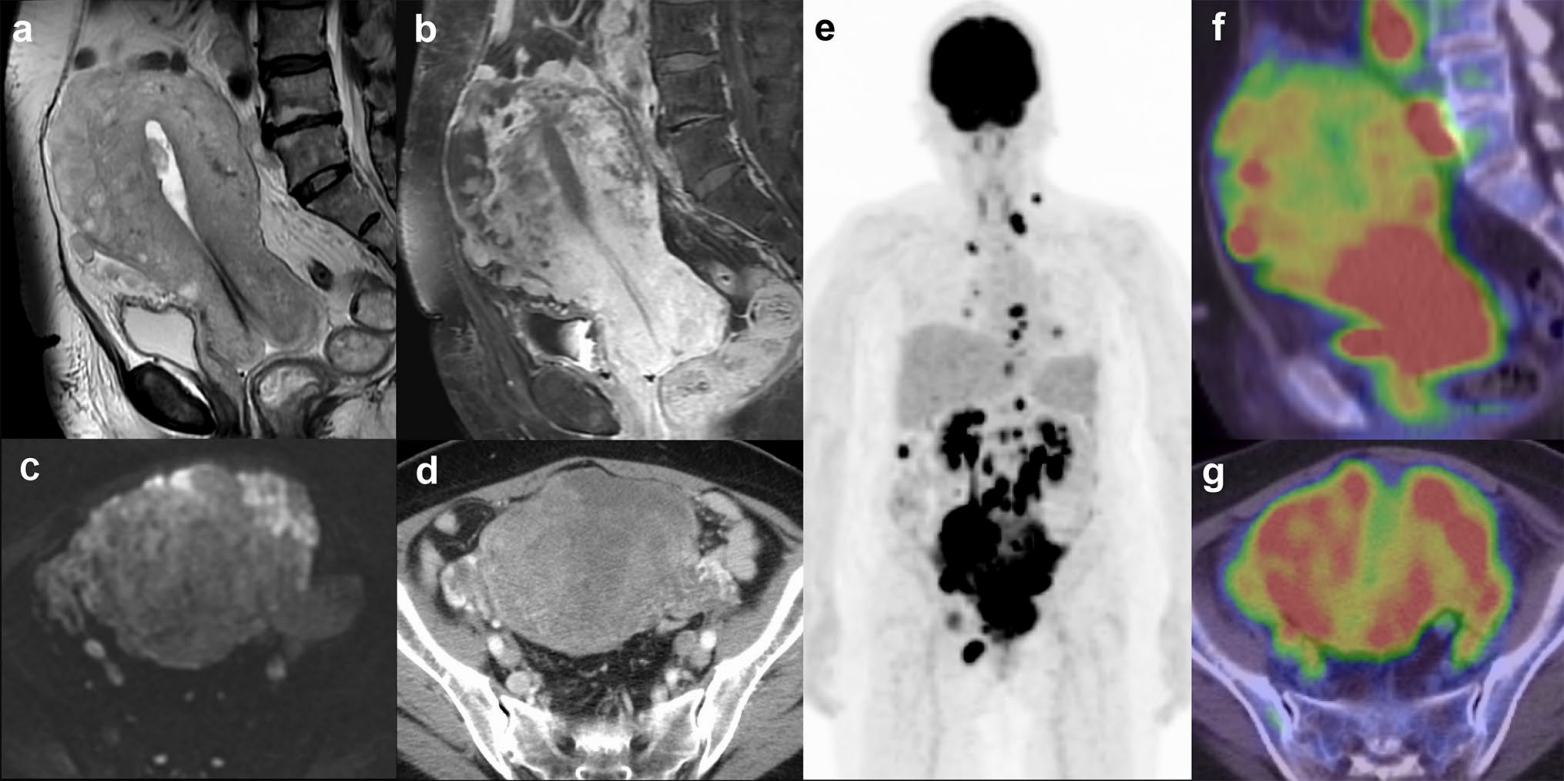
A case of a 61-year-old woman with dedifferentiated endometrial carcinoma (no. 10). **a** Sagittal T2-weighted image showing an enlarged uterus with an irregular shape and heterogeneous intensity (diffuse involvement pattern). **b** Sagittal contrast-enhanced T1-weighted image showing heterogeneous enhancement. **c** Diffusion-weighted (b = 1000 s/mm^2^) image showing diffuse high intensity. Endometrial thickening is not observed. **d** Axial contrast-enhanced computed tomography showing an enlarged uterus with heterogeneous enhancement. **e** Maximum intensity projection of positron emission tomography with 2-deoxy-2-[fluorine-18] fluorodeoxyglucose integrated with computed tomography showing a uterine primary carcinoma with multiple metastases from the supraclavicular lymph node to the inguinal lymph node. **f** Sagittal and (**g**) axial fused images showing diffuse high 18F-fluorodeoxyglucose uptake in an enlarged uterus. The maximum standardized uptake value of the primary tumor is 14.12

The median tumor diameter was 53 mm (range: 16–206 mm). All four patients with tumor sizes > 13 cm on MR images died, whereas six of the other seven patients with tumor sizes < 7 cm were alive. Primary tumors were mass-forming lesions located in the endometrium in nine participants (nos. 1–9) (Figs. 1, 2, 3). Tumors were located in the upper part in three, middle part in one, lower part in one, and entire endometrial cavity in four patients. The remaining two participants (no. 10 and 11) presented with an enlarged myometrium, indicating diffuse involvement of tumor cells (Fig. 4). In these two patients, the uterus was enlarged without endometrial thickening and the borderline of the junctional zone could not be identified on MRI. Tumor extension into the myometrium was observed in eight of the nine patients with primary tumors in the endometrium and was limited to the endometrium in one patient. Extension of the cervical stroma was absent in seven patients and present in four.

Of the nine patients in whom T1WIs were acquired, eight showed iso-signal intensity and one showed slightly high signal intensity relative to the myometrium or skeletal muscle; seven patients showed partially high signal intensity. On T2WIs, tumors in all the patients were hypointense relative to the normal endometrial regions and most tumors showed high signal intensity relative to the myometrium or skeletal muscle; tumors in six patients showed partially low signal intensity. Tumors in three patients showed homogeneity and in seven patients showed heterogeneous signal intensity. All eight patients in whom DWI was performed showed high signal intensity; furthermore, six of the eight patients in whom CE-WI was performed showed heterogeneous enhancement. Four of the eight tumors showed partially nonenhanced areas. Three patients showed areas of strong enhancement on CE-T1WIs.

### Pathological findings

Pathological findings are summarized in Table 2. Hemorrhage was present in all tumors: microscopically visible only (1 +) in one and macroscopically < 25% (2 +) in five, 23–50% (3 +) in one, and > 50% (4 +) in one patients. Necrosis was also present in all patients: microscopically visible only (1 +) in one and macroscopically < 25% (2 +) in three patients, 25–50% (3 +) in one patient, and > 50% (4 +) in three patients. The approximate proportion of undifferentiated components in the tumors ranged from 20% to > 99% (median, 90%).

### Correlation of MRI findings with pathological findings

The MRI and pathological findings were compared in eight of the specimens obtained at surgery, except one patient in which the tumor had resolved with preoperative chemotherapy. Of the seven patients with macroscopic hemorrhages (≥ 2 +), six had a partially high signal on T1WI or a partially low signal on T2WI. Of the seven patients with macroscopic necrosis (≥ 2 +), three with tumor diameters > 5 cm showed areas that were not enhanced on CE-T1WI. All eight patients had hemorrhage or necrosis and six had a heterogeneous signal intensity on T2WI. Of the seven patients who underwent CE-T1WI, five showed heterogeneous enhancement.

## Discussion

DEC is a rare and aggressive high-grade malignancy characterized by the co-existence of UC and low-grade EmC. DEC was reclassified in the World Health Organization’s 2014 Classification of Tumors of the Female Reproductive Organs [1].

DEC has recently garnered a lot of clinical and pathological attention with respect to cancer gene therapy. Recent studies have shown that the UC component in DEC is associated with the loss of MMR protein [7], suggesting an association between DEC and Lynch syndrome [1]. Some studies have evaluated MRI findings in patients with endometrial carcinoma with MMR deficiency [8], and genetic mutations causing MMR deficiency are also attracting attention in the field of radiology.

We determined that MRI findings in the uterus can be used to tentatively classify tumors into two patterns according to the shape of their development: typical mass-forming lesions with or without myometrial extension and diffuse involvement of an enlarged uterus without endometrial thickening. The appearance of a diffuse enlarged uterus on MRI is associated with a poor prognosis because of the inoperable state of this condition at the time of diagnosis performed via needle biopsy. However, small-sized mass-forming lesions in the endometrium have a relatively better prognosis because such tumors can often be completely resected. Even with mass-forming tumors, the prognosis tended to be worse for tumors sized ≥ 13 cm.

The mass-forming type resembles the most common imaging findings of endometrial carcinoma. On MRI, the masses are usually exophytic with or without myometrial extension; endometrial lesions are usually seen as low-to-isointense masses on T1WI and as masses with intermediate signal intensity lower than that of the normal endometrium on T2WI. On dynamic CE-MRI, endometrial carcinoma appears less enhanced compared with the myometrium [9]. Compared with adjacent tissues, tumors typically exhibit restricted diffusion, which is seen as an area of high signal intensity on DWI and an area of hypointensity on apparent diffusion coefficient maps [10]. Carcinosarcomas are also heterogeneous tumors with areas of hemorrhage and necrosis and enhance more avidly than the adjacent normal myometrium [11] and present a similar appearance to the mass-forming type. Conversely, diffuse involvement types develop endophytically and invade the myometrium diffusely, causing uterus enlargement. These findings are similar to those of malignant lymphomas, metastatic tumors, and high-grade endometrial carcinomas (such as neuroendocrine carcinomas), which have been reported to present with similar uterine enlargement [12–14]. In addition, tumor location varied in our study, although one study has reported the tumor to be more commonly present in the lower uterine segment [4].

In a previous study, Han et al. [5] reported pathological findings of DEC located in the endometrial cavity based on CT and MRI images of four patients. Three patients showed high signal intensity on T2WIs and heterogeneous enhancement on CE-T1WIs. These imaging findings were similar to those of the mass-forming lesions described in the present study. Regarding pathological findings, some studies have reported the primary tumor of DEC as a polypoid or bulging mass with necrotic areas in the endometrial bodies, similar to the mass-forming lesion [4, 5, 15–21]. We did not find any reports of diffuse myometrial uterine involvement of DEC, as in the present study, in the literature.

We compared MRI and pathological findings of eight patients from whom surgical specimens were obtained. Most patients with macroscopic hemorrhage showed a partially high signal intensity on T1WI or a partially low signal intensity on T2WI. Intratumoral hemorrhage may be reflected on MRI. Among the patients with macroscopic necrosis, only those with a tumor size of > 5 cm showed a nonenhanced area on CE-T1WIs. These patients had > 25% necrosis within the tumor volume. If the tumor was large and necrosis was extensive, it could be recognized as a nonenhanced area. It would have been difficult to recognize nonenhanced areas in tumors < 5 cm and with < 25% necrosis. The heterogeneous signal on T2WI and heterogeneous enhancement may reflect a substantial degeneration of these hemorrhagic and necrotic areas.

The undifferentiated component was extensive in most cases. In the case shown in Fig. 2 (no. 3, UC component = 70%), the strongly enhanced areas on CE-T1WIs were consistent with undifferentiated components; however, as shown in Fig. 1 (no. 4, UC component = 90%), it was impossible to distinguish between UC and EmC components of the tumor in most cases. As shown in Fig. 3 (no. 8), > 99% of the area comprised undifferentiated components, although the enhancement was weak. This may be due to the mixture of extensive necrosis, as shown by the microscopic findings; these findings suggest that small tumors would have strong enhancement and large tumors would be associated with marked necrosis and hemorrhage.

In most cases, undifferentiated components accounted for a large proportion of the tumors and did not correlate with prognosis. However, one patient (no. 2) with < 20% undifferentiated components had no metastases and a good prognosis. There is a possibility of a better prognosis if the percentage of undifferentiated components is low.

These features on MR images do not provide a clear distinction from other endometrial carcinomas or carcinosarcomas, particularly in the mass-forming type of DEC. However, needle biopsies often lead to suspected malignant lymphoma pathologically. Hence, it is important for radiologists to know about DEC.

Our study had several limitations which should be acknowledged. First, the number of cases is small due to the rarity of DEC. Second, this is a retrospective study. The MRI scanners, devices, sequences, and imaging parameters were not uniform because of the retrospective nature of the study. However, we interpreted MRI findings visually and reduced the quantitative difference. Thus, we believe that this point might not have influenced our results. Third, three cases were diagnosed morphologically and immunohistologically by needle biopsy. Therefore, the whole tumor of these cases could not be evaluated pathologically. Even in cases where the biopsy specimen contained only a UC component, our case with a hysterectomy specimen showed a small amount of low-grade EmC components in all of the surgically resected tumors sampled extensively. Based on the concept that the biopsy diagnosis is an estimation of the histological type by observation of a limited part of the tumor, we considered them to be suggestive of DEC/UC.

## Conclusion

The MRI findings of DEC showed two patterns: mass-forming lesions in the endometrium and diffuse involvement of an enlarged myometrium. The prognosis of the diffuse myometrial type was poorer than that of the mass-forming type. Most patients with DEC had intratumoral hemorrhages, and the hemorrhagic areas showed high signal intensity on T1WI or low signal intensity on T2WI. Additionally, large tumors (> 5 cm) had gross necrosis, which appeared as nonenhanced areas on CE-MRI.
